# Infectious Zika virus in vaginal secretions from an HIV-infected woman, France, August 2016

**DOI:** 10.2807/1560-7917.ES.2017.22.3.30444

**Published:** 2017-01-19

**Authors:** Pauline Penot, Ségolène Brichler, Jean Guilleminot, Caroline Lascoux-Combe, Olivier Taulera, Emmanuel Gordien, Isabelle Leparc-Goffart, Jean-Michel Molina

**Affiliations:** 1 Internal Medicine and Infectious Diseases Department, Andre Gregoire Hospital, Montreuil, France; 2 Infectious and Tropical Diseases Department, Saint Louis Hospital, Paris, France; 3 Clinical Microbiology Department, Avicenne Hospital, Bobigny, France; 4 Family Planning Clinic, Saint Louis Hospital, Paris, France; 5 Internal Medicine Department, Saint Louis hospital, Paris, France; 6 Institut de Recherche Biomédicale des Armées, National Reference Laboratory for arboviruses, Marseille, France; 7 UMR ‘Emergence des Pathologies Virales’ (EPV Aix Marseille university, IRD French Institute of Research for Development, INSERM French National Institute of Health and Medical Research, EHESP French School of Public Health), Marseille, France; 8 These authors contributed equally to this work

**Keywords:** Zika virus, imported viral disease, vector-borne infections, sexually transmitted infections

## Abstract

A woman with controlled HIV infection developed in late August 2016 a pruritic rash with fever and conjunctival hyperaemia after a trip to the French Caribbean islands. On day 3 after symptom onset, Zika virus RNA was detected in plasma, urine and vaginal samples with respective viral loads of 3.8, 6.1 and 5.3 log copies/mL. Notably, we demonstrated the presence of infectious Zika virus particles in the vaginal samples by isolation in cell culture.

A female patient returned to France from the Caribbean islands with acute Zika virus infection. After obtaining informed consent, we collected vaginal swabs for both RNA detection and virus culture in order to investigate the infectivity of vaginal secretions.

## Case presentation

A French woman in her 40s, with a controlled HIV-1 infection, travelled to French Caribbean islands (Martinique for seven days then Guadeloupe for three weeks) in summer 2016. On the day of return to France, she felt asthenic with myalgia. Two days later, a pruritic rash appeared on her face, chest, back and arms, with abdominal pain and diarrhoea. She consulted her general practitioner, who referred her to our department on the same day.

Her medical history was limited to a virologically suppressed HIV-1 infection with CD4^+^ T-cell count over 500/mm^3^ under antiretroviral therapy (emtricitabine/tenofovir and nevirapine). Physical examination showed a widespread itching maculopapular exanthema, conjunctival hyperaemia and mild fever (38.1 °C). Intense asthenia and diffuse myalgia were still ongoing.

As the symptoms were consistent with acute Zika virus infection, the patient consented to have a vaginal swab, after being informed that there would be no direct benefit from knowing the test result. In the absence of a standardised collection protocol, vaginal secretions were collected by direct swab (vaginal sample 1) and after instilling 5 mL of saline solution between the cervix and the posterior vaginal wall (vaginal sample 2). At her request, her scheduled cervical Pap smear was performed at the same time.

Standard laboratory tests showed moderate lymphocytopenia with 1,120 cells/mm^3^ (norm: > 1,200/mm^3^) and C-reactive protein 8 mg/L (norm: < 5 mg/L). RT-PCR assays were negative for dengue and chikungunya viruses in plasma (Fast-Track Diagnostics, Luxembourg) and a test for NS1 antigen for dengue virus was also negative (Bio-Rad, Marne la Coquette, France). Detection of Zika virus RNA by RT-PCR (Altona Diagnostics, Hamburg, Germany) was positive in plasma, urine and vaginal secretions, three days after onset of symptoms. The viral RNA load was 3.8 log copies/mL in plasma, 6.1 in urine, 5.3 in vaginal sample 1 and 3.9 in vaginal sample 2. The use of a saline solution instillation for the second sampling possibly led to a dilution of the sample. The two vaginal samples were inoculated on Vero and C6/36 cells, and Zika virus was isolated from both, demonstrating the presence of infective Zika virus. All methods were performed as detailed in the supplementary data of a previous publication [1]. According to French guidelines, no serological analysis was performed for dengue and Zika viruses. 

The patient’s fever subsided on day 3, and the rash on day 6 after symptom onset. A transient bilateral knee pain occurred on day 4.

The patient consented to a second genital swab 10 days after the onset of symptoms: no viral RNA was detected in vaginal secretions and in cervical mucus. No serum collection was performed during the second visit. At that time, she had recovered from all symptoms except a mild persistent asthenia. Her husband did not present symptoms evocative of Zika virus infection during or after the journey. The patient stated that their sexual intercourses were always protected by condom use. 

## Background

Initially, Zika virus was thought to be exclusively transmitted through mosquitoes, but sexual transmission was subsequently reported. Transmission from men to their female or male sexual partners has been well and often demonstrated and has been observed up to 44 days after symptom onset [2-5], whereas only one case report suggests a possible woman-to-man transmission, which was concurrent with the acute infection symptoms in the female partner [6]. Three teams have reported the presence of Zika virus RNA in female genital secretions up to 14 days after symptoms onset, but Zika virus infectivity was not proven [7-9]. None of the women with symptomatic Zika infection involved in an assisted reproductive technology programme on Guadeloupe had detectable Zika virus in their genital tract beyond the second week of follow up, despite regular monitoring by Zika RT-PCR for up to 3 months [7].

## Discussion

This seldom reported and transient presence of Zika virus in the female genital tract contrasts with an extensive literature about its persistence in semen, where RNA has been detected up to six months after return from endemic areas [4,10-14]. Moreover, Zika virus from semen has been isolated in cell culture in four patients, up to 69 days after onset of symptoms, but had never been isolated from the human female genital secretions before [1,15-17]. 

The short time period during which we could detect Zika virus in our patient’s genital tract is consistent with our prior failure to detect the virus by RT-PCR from vaginal swabs in two other patients, seven and eight days after the onset of symptoms [18]. Although viral load was higher in our patient’s vaginal secretions than in her serum, it remained lower than what could be measured in some semen samples [1,15,16]. Our patient had been living with virologically HIV suppressed infection for years and had an almost normal lymphocyte cell count. It is unlikely that her HIV-infection influenced the evolution of her Zika virus infection, especially as the clinical presentation and evolution were similar to those observed in HIV-negative patients. 

Recently, a mouse model of Zika virus infection by vaginal exposure demonstrated that Zika virus replicated within the genital mucosa and could lead to a fetal infection [19], but no data has been available up to now on the infectiousness of a vaginally situated virus in humans. Our findings suggest a short period of infectivity of women with acute Zika virus infection through their genital secretions. This short duration of virus shedding in genital secretion may explain why to date only one case of female to male transmission has been reported. Yet, it remains unknown whether Zika virus can establish a reservoir in the female genital tract and infect follicles and/or ovules.

Recent evidence of extended presence of the virus in semen and of possible transmission from women to men has led to an update of the United States Centers for Disease Control and Prevention (CDC)’s guidance on the prevention of sexual transmission of Zika virus [20]. Current French guidelines and the CDC recommend a deferral period of at least 2 months before women returning from an area with circulating Zika virus can access medically assisted reproductive technology programmes. When this delay cannot be adhered to (e.g. fertility preservation before chemotherapy), testing vaginal samples by RT-PCR should be recommended.
